# Supplementary material for: Challenges in the distribution of antimicrobial medications in community dispensaries in Accra, Ghana
Source: PLoS One. 2024 May 29;19(5):e0281699. doi: 10.1371/journal.pone.0281699 (PMC11135707; doi:10.1371/journal.pone.0281699)
Supplement: S1 File — (PDF) [file pone.0281699.s001.pdf]

S1 File: Challenges in the distribution of antimicrobial medications in community dispensaries in Accra, Ghana

May 15, 2024

Hannah Camille Greene[1], Kinga Makovi[1], Rafiatu Abdul-Mumin[2], Akhil Bansal[3], Jemima A. Frimpong[1*]

1 Social Science Division, New York University Abu Dhabi, Abu Dhabi, United Arab Emirates
2 Department of Biochemistry and Forensic Sciences, School of Chemical and Biochemical Sciences, C.K Tedam University of Technology & Applied Sciences, Navrongo, Ghana
3 Faculty of Medicine and Health, University of Sydney, Sydney, Australia
[*] To whom correspondence should be addressed: Jemima Frimpong, jafrimpong@nyu.edu

Table S1. Challenges by category and subcategory with definitions and examples.

Code/subcode	Definition	Example
**Entity**		
Low sales	Low circulation in general at the shop level	"This area [is] not very lucrative, doesn't get a lot of people to buy"
Competition	Competition from other shops	"Too many pharmacies. Competition proximity... so if not in very good position, [customers] would not come to you"
Workplace	Boredom/money issues of the respondent	"It gets boring sometimes. It gets so boring doing the same thing over and over again."
**Customer**		
Attitude	Rude customers	"Customers wanting to argue; people think you are trying to patronize them instead when you want to give them the good treatment."
Self-medication	Customers wanting to self-medicate	"People coming in with self-diagnoses and the medications they want, sometimes without considering your recommendations or opinions"
Treatment	Issues with the correct course of treatment (facing complicated symptoms, serious illness, or disagreements about diagnosis)	"Facing difficult cases of illnesses that I cannot help with"
Adherence	Taking too much or too little, or not on the prescribed regimen	"To convince to take for the 3 days course"
**Community**		
Payment	Money problems	"Customers wanting to buy on credit"
Education	Lack of education of customers (not understanding how antibiotics work, or active ingredients etc.)	"Some patients don't understand antibiotics for instance. They say 'I am buying, don't understand why you don't want to sell it to me."'
Language barrier	Language barrier	"Language barrier. [I] cannot speak Hausa language properly"
Diagnostic testing	Lack of diagnostic testing available	"Availability and accessibility of diagnostic tests and diagnostic facilities to customers/patients"
**Global**		
Brand	Insisting on international brands; this could originate from word-of-mouth, advertising etc.	"To convince them to take it. Let's say they know Arfan and you don't have Arfan, you have a different company's one. [It's difficult] to convince them to buy the same medication, [even at] same strength"
Supply chain	Not getting the medicines customers want, or getting it near the expiry date	"In my shop for instance, I think probably the lead time of the medications. And actually sometimes the drugs you request that you want, they don't really come"
**Other**		
Other	Idiosyncratic reason	"Clarity of prescriptions from doctors. Handwriting, clarity of dosage; sometimes [a medicine is] supposed to be taken twice a day but doctor has written once a day"
**None**		
None	No challenges	"Everything seems to be normal for me. I enjoy doing this work"

The survey questionnaire was used as the research instrument.

Introduction

Informed Consent. Thank you for agreeing to take part in this survey! The purpose of the research is to study patterns in the use of antibiotics and antimalarials in different parts of Accra, and to study how different characteristics of customers and neighborhoods could predict which antibiotics and antimalarials are available.

Things you should know:

● If you choose to participate, you will be asked a series of questions about your background, your role here, and your experiences selling antibiotics and antimalarials. The researcher will record the answers you give. This will take up to approximately one hour.

● Participation is completely voluntary, and you are welcome to withdraw at any time. The survey can be stopped any time when customers enter so that it doesn't interrupt your business.

● If there are any questions you are not comfortable answering, please say so and the question will be skipped so you don't have to answer.

● If there is anything you don't understand, please ask and it will be clarified. Taking part in this research project is voluntary. You do not have to participate and you can stop at any time. Please take time to read this entire form and ask questions before deciding whether to take part in this research project.

2. PURPOSE OF THIS STUDY

The purpose of the study is to study patterns in the use of antibiotics and antimalarials in different parts of Accra, to see which antibiotics/antimalarials are used most, and to study how different characteristics of customers and neighborhoods could predict which antibiotics/antimalarials are available.

3. INFORMATION ABOUT STUDY RISKS

3.1 What risks will I face by taking part in the study?

Because this study collects information about you, the primary risk of this research is a loss of confidentiality.

3.2 How will the researchers protect my information?
Your name or contact location will not be recorded. Only information about the entire research population will be shared, not connected to your individual identity. Information about this shop will be kept confidential and only disclosed in aggregate, meaning that it will be mixed in with all the other locations so that it isn't traced back to you.

4. INFORMATION ABOUT STUDY BENEFITS

4.1 What will happen to the information collected in this study?
We will keep the information we collect during the research for future research projects/for study recordkeeping. The researchers may visit this location again as part of this project, but we will not record your name or other information that can identify you directly.

4.2 How could I benefit if I take part in this study? How could others benefit?
There are no direct benefits of your participation. However, others may benefit from the knowledge gained from this study through long-term improved access to healthcare.

Student Investigator: Hannah Greene
Email: hcg256@nyu.edu
Phone: +233 059 644 2416

Faculty Advisor: Jemima A. Frimpong
Email: jafrimpong@nyu.edu

Consent.
Consent to Participate in the Research Study

By checking off "yes," you are agreeing to be in this study. Make sure you understand what the study is about before you sign. I/We will give you a copy of the informed consent information document for your records and I/we will keep a copy with the study records. If you have any questions about the study after you sign this document, you can contact the study team using the information provided above.

I understand what the study is about and my questions so far have been answered.
I agree to take part in this study.

- ○ Yes
- ○ No

Pharmacy or chemical shop?

place_type. Type of place

- ○ Pharmacy
- ○ Chemical shop
- ○ ______ Other

Survey info

Day. Day of the week

- ☐ Monday
- ☐ Tuesday
- ☐ Wednesday
- ☐ Thursday
- ☐ Friday
- ☐ Saturday
- ☐ Sunday

Q1 Age. How old are you?

- ○ Under 18
- ○ 18-24 years old
- ○ 25-34 years old
- ○ 35-44 years old
- ○ 45-54 years old
- ○ 55-64 years old
- ○ 65+ years old

Q2 Gender. Gender

- ○ Male
- ○ Female
- ○ [ ]

Q3 startyear. What year did you start working here?

[ ]

Q4 found_job. How did you find this job? (short answer, general response - for example, working for family business, applied at headquarters, applied online, referred by friend)

[ ]

Typical sales

Q5 num_cust. Typical day

How many customers do you typically see in a shift? (number of customers / people who enter shop each day you work here)	
How many of them buy medications? (number)	
other info	

Q6 popular_prod. What are the most popular products you sell? (enter one or more; can be any kind of products, medication or not)

1.	
2.	
3.	
4.	
5.	

Q7 sales_fraction. How much of your sales are these: (in fraction and/or percentage) (if they give a word answer, write it in the "fraction" box. Write fraction as the number of purchasers out of the number of customers/medicine purchasers listed above)

Antibiotics (%)	
Antibiotics (fraction)	
Antimalarials (%)	
Antimalarials (fraction)	

Respondent Information

Q8 Role. Role

- ☐ Pharmacist
- ☐ Shop owner
- ☐ Medical Counter Assistant
- ☐ ______ Other employee

Q9 days_work. How many days a week do you work here?

	0	1	2	3	4	5	6	7
Number of days								

Q10 shift_info. Shop information

How many hours a day is the shop open?	
How many total employees work here?	
How many hours are your shifts? (length in hours)	
other work information (anything else relevant)	

Q11 Training. Do you have any specific training/qualifications for selling medicines?

duration in months (4 years = 48 months, 6 years = 60 months)	

type of training (i.e. Bachelor of Pharmacy, MCA/Medical Counter Assistant) [ ]

edu_level. What is your highest level of education completed?

- [ ] No formal education
- [ ] Primary school (up to year ___)
- [ ] JSS / Junior secondary school (up to year ___)
- [ ] SHS / Senior high school (up to year ____)
- [ ] Bachelor's or higher
- [ ] Other

Pharmaceutical Antibiotics

Q12 AB_available.
What types of antibiotics do you sell and in what form? (check off all which are sold here)

	Capsule	Liquid (suspension or syrup)	Injection	Tablet	unknown
Flucloxacillin	☐	☐	☐	☐	☐
Cefuroxime	☐	☐	☐	☐	☐
Ciprofloxacin	☐	☐	☐	☐	☐
Amoxicillin	☐	☐	☐	☐	☐
Ceftriaxone	☐	☐	☐	☐	☐
other [ ]	☐	☐	☐	☐	☐
other_2 [ ]	☐	☐	☐	☐	☐

Q13 most_sold_AB.
Which of these antibiotics do you sell most often? (drag and drop into box for rank: most often, sometimes, rarely, or never)

Items	most often
Flucloxacillin	
Cefuroxime	
Ciprofloxacin	
Amoxicillin	
Amoxicillin-clavulanic acid	
Ceftriaxone	
other	

sometimes

rarely

never

Q14 antibiotic_info. Antibiotic information: answer only for the most often sold antibiotics. (can answer multiple brands, list in commas within the box)

	Brand	How many days is the recommended treatment course length?	How many

	local/generic	originator/imported Brand brand	local/generic	How many days is the recommended treatment course length? originator/imported brand	local/generic How many

	local/generic	originator/imported brand	local/generic	originator/imported brand	local/generic
Flucloxacillin					
Cefuroxime					
Ciprofloxacin					
Amoxicillin					
Amoxicillin-clavulanic acid					
Ceftriaxone					

Q15 antibiotics_sold. Antibiotic information: answer only for the most often sold antibiotics

	In the last 7 days, how many patients bought this?	How many capsules did each customer buy (average)?	Today, how many patients bought this?	How many capsules did each customer buy (average)?
Flucloxacillin				
Cefuroxime				
Ciprofloxacin				
Amoxicillin				
Amoxicillin-clavulanic acid				
Ceftriaxone				

Q16 entire_course . Compared with your recommendation or their prescriptions, how often do people buy the whole treatment course of antibiotics (antibiotics if pharmacy, or antimalarials if chemical shop)?

- ○ Always
- ○ Most of the time

- ○ ☐ About half the time
- ○ ☐ Sometimes
- ○ ☐ Never

Q17 expect_adhere. Do you think they actually take the whole treatment course of antibiotics? (or antimalarials if chemical shop)

- ○ ☐ Definitely yes
- ○ ☐ Probably yes
- ○ ☐ Might or might not
- ○ ☐ Probably not
- ○ ☐ Definitely not

Liquids - injections, suspensions, or syrups

Q18 liquid_units. What units do you use when giving/advising how much to take for liquid medicines? (suspensions, syrups)

- ☐ mL
- ☐ teaspoon
- ☐ cc
- ☐ ☐ other

Q19 measuring_cup. Do liquid medicines come with a measuring cup?

- ☐ ☐ never
- ☐ ☐ sometimes
- ☐ ☐ always

Q20 Injections. Do you do any injections here?

- ○ yes
- ○ [ ] no, but we sell the vials
- ○ [ ] no, we do not sell the vials either

Antimalarials (AM)

Q31 AM_sales_freq. How often do you sell antimalarials? (enter any more specific information in the box like "every 2-3 days")

- ☐ [ ] daily
- ☐ [ ] weekly
- ☐ [ ] monthly
- ☐ [ ] other

Q32 AM_most_sold. What type of antimalarials do you sell most?

[ ]

Q33 AM_available. Which antimalarials do you sell here?

Items

dihydroartemisinin-piperaquine (DHAP)

artemether–lumefantrine (AL)

artesunate–amodiaquine (AA)

most often

sometimes

never

Q34 antimalarialinfo. Antimalarial types (answer for whichever are sold frequently)

	Brand(s)		Cost per treatment course		
	local/generic	originator/imported	local/generic	originator/imported	lo[illegible]
dihydroartemisinin-piperaquine (DHAP)					
artemether–lumefantrine (AL)					
artesunate–amodiaquine (AA)					
artesunate-sulfadoxine-pyrimethamine (ASP)					
other					

Q35 malaria_tests. Do you do malaria tests here?

- ○ No
- ○ Yes
- ○ other info

Q36 AM_requests. If people think they have malaria, do they request a specific antimalarial / type of medication?

- ○ Never
- ○ Sometimes
- ○ Often

Q37 AM_preferred. Which one?

- ☐ DHAP (Dihydroartemisinin-piperaquine)
- ☐ AL (not specific brand, artemether-lumefantrine)
- ☐ Lufart (AL)
- ☐ Lonart (AL)
- ☐ Coartem (AL)
- ☐ AA (Amodiaquine-artesunate)
- ☐ other

Q38 cust_concerns. What are some concerns expressed by customers for antibiotics or antimalarials?

- ☐ Dosage
- ☐ Cost
- ☐ Side effects
- ☐ Efficacy (if they won't work)
- ☐ other

Q39 AM_cheapest. Which antimalarials are the cheapest?

- ☐ DHAP
- ☐ AL
- ☐ Lufart

- [ ] Lunart
- [ ] Coartem
- [ ] ____ AA
- [ ] ____ other

Q40 AM_effective. Which antimalarials do you think are the most effective?

- [ ] all are equally effective
- [ ] ____ DHAP
- [ ] ____ AL
- [ ] ____ Lufart
- [ ] ____ Lunart
- [ ] ____ Coartem
- [ ] ____ AA
- [ ] ____ other

Prescriptions, requests, and payments for medications

Q41 Rx_numbers. Prescriptions:

When someone comes in with a prescription, approximately how many medicines have they usually been prescribed at once? (How many different medications are usually listed with one prescription?)	
Is there seasonal variation in the number of prescription medications? Does the number of patients with prescriptions, or the number of prescriptions per patient, seem to be higher at different times of year?	
other info	

Q42 cannot_pay. What happens if someone has a prescription **but cannot pay for the recommended amount / type of antibiotics or antimalarials?**

- ☐ offer cheaper brand
- ☐ offer smaller quantity
- ☐ offer different type of medication
- ☐ offer credit / payment later
- ☐ other

Q43 freq_request. How often do people come in and ask for/point to/request specific medicines? (when people do not have prescriptions)

- ○ Never
- ○ Rarely
- ○ Sometimes
- ○ Often

Q44 most_requested. What is the most common medication people would walk in and request?

Q45 AB_advising. What happens if someone does not have a prescription for any antibiotics but you think they need them?

Q46 alternatives. How often does it happen that a person intending to buy antibiotics: (or antimalarials if chemical shop)

	never	rarely	sometimes	often
buys a partial course	○	○	○	○
decides not to buy anything at all	○	○	○	○

	never	rarely	sometimes	often
chooses a different medication [ ]	○	○	○	○
other [ ]	○	○	○	○

Q47 patient_stress. Financing medications: rank average patient stress about paying for the antibiotics or antimalarials

	1 no problem at all	2 mild concern	3 middle	4 significant concern	5 extremely expensive, large financial burden
Rank average patient	○	○	○	○	○

Q48 methods_payment. What are the available methods of paying for medications?

	Available?<br>check if available	How commonly used?<br>how often is this used?
Cash [ ]	☐	[ ]
Mobile Money (MoMo) [ ]	☐	[ ]
Card / POS [ ]	☐	[ ]
Insurance - NHIS [ ]	☐	[ ]
Insurance - private [ ]	☐	[ ]
Credit (return and pay another day) [ ]	☐	[ ]

Q49 cust_repeat. Do most customers come repeatedly, or just once? Do they return many times / do you recognize customers?

- ☐ [ ] Most come one time only
- ☐ [ ] Some return

- [ ] ____ Most come many times

Q50 local_cust. Do customers seem to come mostly from this neighborhood or outside?

- [ ] ____ live in this neighborhood
- [ ] ____ visit from other neighborhoods
- [ ] ____ work in this neighborhood
- [ ] ____ other

Q51 cust_transport. How do customers seem to get here?

- [ ] walk
- [ ] tro tro
- [ ] motorbike
- [ ] car
- [ ] taxi
- [ ] Bolt/Uber
- [ ] ____ other

Supply chain

Q52 supply_delay. Is there a delay between when you run out of an antimalarial or antibiotic and when you get a new supply?

- [ ] ____ no delay
- [ ] ____ days
- [ ] ____ weeks
- [ ] ____ months

Q53 wholesaler. Do you know the source of where the antimalarials / antibiotics come from wholesale?

Perceptions of customers

Q54 cust_age. Most common / estimated average age of customers who enter the shop

- ☐ Under 18
- ☐ 18 - 29
- ☐ 30 - 49
- ☐ 50 or older
- ☐ other

Q55 cust_gender. What proportion of clients are female?

	0	10	20	30	40	50	60	70	80	90	100
% who are female											

Q56 others_buying. Who do you think they are usually buying antibiotics/antimalarials for?

- ☐ self
- ☐ spouse
- ☐ child
- ☐ parent
- ☐ other family member
- ☐ other

Q57 symptom_aware. Are you often aware of the symptoms of the patient?

- ☐ Always

- ☐ Most of the time
- ☐ About half the time
- ☐ Sometimes
- ☐ Never

Q58 common_sympt. What are the most common symptoms people come with?

	Check if one of the most common<br>Check	notes<br>other info
Fever	☐	
Diarrhea	☐	
Cough	☐	
Sore throat	☐	
Vomiting	☐	
Headache	☐	
Stomach upset	☐	
Body aches	☐	
other	☐	

Q59 Rx_symptoms. If they come with a prescription, how often are you aware of the symptoms of the patient?

- ☐ Often
- ☐ Sometimes
- ☐ Never

Q60 severity. Symptom severity

1 very mild	2 mild	3 medium	4 severe	5 very severe

	1 very mild	2 mild	3 medium	4 severe	5 very severe
How severe do symptoms seem to be on average?	○	○	○	○	○

Neighborhood information

Q61 Neighborhood. Shop neighborhood

- ☐ Labone
- ☐ Cantonments
- ☐ Osu
- ☐ La
- ☐ East Legon
- ☐ James Town
- ☐ Korle Gonno
- ☐ Nima
- ☐ Agbogbloshie
- ☐ ________ other

Q62 neighbor_income. Rank income level of this neighborhood

- ☐ Low
- ☐ Medium
- ☐ High
- ☐ ________ other

Q63 household_size. How many people live in an average household in the neighborhood?

- ☐ ________ unknown - do not live here
- ☐ ________ live here - known
- ☐ ________ unsure - estimate

Q64 water. Most common source of drinking water in this neighborhood

- ☐ ______ Pipe in home
- ☐ ______ Pipe or well outside dwelling
- ☐ ______ Natural sources (river/stream, dugout)
- ☐ ______ Sachet/bottled
- ☐ ______ Water trucks & vendors

Q65 toilets. Most common type of toilet for this neighborhood

- ☐ ______ Flush toilet in house
- ☐ ______ Pit latrine in house
- ☐ ______ KVIP (Kumasi ventilated improved pit)
- ☐ ______ Toilet in another house
- ☐ ______ Public toilet
- ☐ ______ No toilet facility (bush/beach)

Q66 lighting. Lighting

- ☐ ______ Electricity
- ☐ Kerosene
- ☐ Candle/torch
- ☐ None
- ☐ ______ other

Q67 house_type. Type of houses in this neighborhood

- ☐ Aluminum walls
- ☐ Cinderblock walls
- ☐ Drywall
- ☐ Apartment buildings

☐ ______ other

Challenge

Q68 challenge_faced. What is the biggest challenge/concern/difficulty you face as a medicine seller?

______

Inventory records

Q70 logbook_consent. Does your employer or the shop owner consent to allow the researcher to photograph the record book of your sales/inventory? (This can be for any dates of your choosing, with any patient information covered up by a piece of paper to remove any identifying information from the photograph)

- ○ ______ Yes
- ○ ______ No
- ○ ______ Did not ask

Q71 cust_tally. Number of customers who entered shop during the survey

______

Q72 survey_length. Time taken for interview (minutes)

______

The End. This is the end of the survey. Click the arrow to submit. Thank you!

Powered by Qualtrics
