# Supplementary material for: Challenges in the distribution of antimicrobial medications in community dispensaries in Accra, Ghana
Source: PLoS One. 2024 May 29;19(5):e0281699. doi: 10.1371/journal.pone.0281699 (PMC11135707; doi:10.1371/journal.pone.0281699)
Supplement: Attachment — Submitted filename: Response_to_reviewers.pdf [file pone.0281699.s004.pdf]

We are grateful for the Editors and Reviewers for their patience, and the opportunity they afforded us to improve our work through peer-review. Unfortunately, due to health-related reasons, followed by an unfortunate accident, we were unable to meet the deadline originally set. We apologize for our tardiness.

We took the time to fundamentally rethink our paper based on the comments that we received. Below, we summarize the main points where our revision focused.

The most important changes include:

1. We went back to the socioecological model of public health and modified it following prior literature. Based on reviewer comments we realized that categories we proposed earlier were analytically less clear than we had hoped, and at times contained significant overlap. Following precedent, we adapted the model for the context of our study, and we believe the new categorization is significantly clearer and better explained in our revision.
2. We also noticed some redundancies in our writing, which we have addressed in the current draft. We hope that our efforts to streamline how information is presented, to eliminate redundancies has further enhanced the readability and comprehension of our key points.
3. As suggested by reviewers, we have now disaggregated our descriptive statistics, as well as the main concerns medicine vendors mentioned by shot type: pharmacy vs chemical/herbal shop. This allowed us a richer analysis which we incorporated throughout the paper.

Further below we address each comment one-by-one.

Reviewer #3: Thank you for inviting me to read this revised submission based on previous reviews.

To curb the global threat of AMR, strengthening community antibiotic stewardship could contribute much to rational use of antibiotics, especially in settings where antibiotics are also available without a quality assured medical diagnosis and prescription. As such, it seems a relevant topic to assess challenges of community medicine providers in Accra.

I still have some concerns however. The primary objective was to understand challenges perceived by medicine vendors. It is assumed that if these could be addressed, it would contribute to health of the population. However, this is not a given. First, the challenges identified are beyond quick fixes, related to poverty, knowledge, infrastructure. Second, to improve the health outcome of the population attending these vendors, it is key that a quality assured diagnoses is made (requiring proper training and refresher courses for staff; quality assured diagnostics), that respectful quality care is delivered (again requiring proper training and refresher course, as well as validated guidelines with empiric antibiotic choices based on the best available surveillance data) and that antibiotics which are being sold are quality assured and well stocked. Several of these issues are mentioned in the discussion already (but without a clear pathway or prioritisation to bring about such major investments). Thus, while addressing reported challenges might increase job satisfaction of the staff, and while there is great potential in involving this group as

gatekeepers to improve health and antibiotic use, it remains to be tested to what extent this results in better care and in better use of antibiotics. The authors need to acknowledge this limitation throughout.

We appreciate the observations by the Reviewer. We agree that “quick fixes” would be far and few, but there are some measures that could be taken, like improving knowledge infrastructure, which may be easier than alleviating poverty. We agree that addressing these challenges would in fact contribute to reducing, and ultimately stopping AMR. We further agree with the Reviewer that quality assured diagnosis includes training both in interpretation of diagnoses and service delivery. To acknowledge these points, we now emphasize them further, and expand on this important point in our Discussion section. Although we make recommendations on ways in which the issues can be addressed and alleviated, we are upfront that our study does not aim to systematically prioritize potential solutions or improvements. In addition, estimating a budget for potential solutions is also outside of the scope of our paper. Interventions and analysis of cost-effectiveness of interventions are indeed necessary. Specifically, we add to the manuscript that:

“*While keeping in mind the limitations of this study, we believe that the insights from shopkeepers provide valuable information to improve the quality of care in private medicine dispensaries. The systemic factors leading to the discussed challenges---poverty, lack of education/knowledge, and faltering infrastructure---extend far beyond any quick fixes. However, ameliorating some of the conditions surrounding medicine shops would contribute to more prudent antimicrobial stewardship. These challenges could elucidate some possible interventions to enable quality-assured diagnoses, well-stocked pharmacies, and patient-focused health care. Future studies should aim to prioritize our recommendations, supported by cost-benefit and cost-effectiveness analyses.”*

The paper should provide more details on the population being sampled, also to provide insight in potential selection bias. In particular, it is stated that all community providers were invited, but that saturation was not achieved, with only 80 included. It is essential to provide details on the total number of each provider (pharmacy, MTC, herbal shop, but potentially also other medicine providers, such as churches, traditional healers, (mobile) market sellers, ..) in the areas, and how a selection was made. For the 17 neighbourhoods (selected by convenience): please clarify what is their population, what % of the population of Accra is covered. In the discussion, reflection on how these non-random selections may have biased results needs to be included. Also, please clarify the turn-over of staff: Table 1 states 42.5% less than 2 years in the position; the discussion mentions over 50%.

We appreciate the Reviewer’s suggestions, and agree that the level of data requested by the Reviewer would be of great usefulness. However, we did not conduct a formal census of dispensaries, or collect population-level data. As such, we cannot assess how many pharmacies and medicine shops there are in Accra for each neighborhood. An overwhelming majority of shops have no web presence either, so enumerating them through these methods is also not feasible. The current analysis was conducted as part of intense ethnographic observation in the identified neighborhoods which, as described, were selected to reflect the *diversity* of living conditions in Accra, and maps on the presence of medicine vendors and pharmacies. While selection may

always be an issue, our intended sampling approach, diversity of neighborhoods selected, and very low refusal rate (only three shops refused to participate), assures us that in the context of this research, selection is a minimal issue. We however agree with the Reviewer's sentiment and have explicitly spelled out in the manuscript the extent to which selection bias may be relevant. Additionally, to our knowledge, there is no publicly available neighborhood-level population census that would allow us to further enhance the description of neighborhoods and assess the sizes of populations served.

As for the turn-over rate, these have been reviewed, and harmonized. We appreciate you pointing out this discrepancy.

Furthermore, methodologically it should be clarified why only individual open-ended questionnaires were used to extract perceived challenges, rather than also bring people together with some (curated) focus group discussion to attain validation and more in depth knowledge.

These additional ways to collect data fell outside of the scope of the study, and the time of the fieldwork. We agree that future work should take up the material currently covered to further validate the findings of our work, perhaps following the avenues that the Reviewer suggests. In our Limitation subsection we write:

> *"Further validation of these findings should be undertaken in future studies, by conducting the survey in additional shops, as well as complementing this with focus groups with medicine vendors to aid interpretation."*

It would have been very relevant to have data on actual health seeking behaviour of the populations in the 17 catchment areas (who is attending which providers when; how much health care is actually provided by medicine vendors), but this was presumably beyond the scope of this study. Also, an extension with exit interviews of customers, or the use of mystery patients, could have provided validation of the self-reported dispensing of the vendors. Please consider if this should be included in the recommendations.

We agree this would have been extremely informative, but as the Reviewer intuited, this was beyond the scope of this study. Future work indeed could follow these pathways. We spell this out in the Limitations subsection, where we write (continuing the quote from our response just above):

> *"Exit interviews with customers and mystery patients should verify shopkeepers' assessments. Furthermore, future studies could seek to verify data with multiple employees of each shop."*

Another potential source of bias is related to the timing of the study: only 2 months were selected. Demand for/use of antibiotics and antimalarials tends to be highly seasonal, and also health care seeking behaviour and accessibility can be impacted by seasonality. Please acknowledge and reflect on this limitation as well.

We agree that seasonality is highly important, and have added this limitation. We also note that the survey questionnaire did ask participants about seasonal variation in medication sales, and in

response they reported higher antimalarial demand during the rainy season and higher rates of other diseases during the harmattan season. We now note this in the paper in multiple places, perhaps most importantly in the Limitations subsection:

> *"As reported by participants, disease outbreaks vary seasonally. However, this study was limited to November--December, in between the rainy and harmattan seasons. It thus provides a limited lens into seasonal variation, and reports about antimalarials are based on recall. "*

The 120 reported challenges in the 80 interviews have been grouped in 5 categories. The specific challenges are presented grouped in Figure 1, but otherwise reported in 5 rather long-winding narratives. This is not very accessible, therefore I strongly suggest that each of the narratives is clearly structured with subheadings along the 2-5 subcategories as presented in Figure 1.

We completely agree with the Reviewer's assessment, and this is one of the major points we emphasized that guided our revision of the paper. We have added subheadings, and streamlined narratives so that they are concise. We have also adapted the socioecological model of public health to our context, and we believe that this has tremendously enhanced our discussion of challenges and streamlined the presentation of our findings. These changes are evident throughout the entire Results section.

Antibiotic use is the result of a demand from customers and supply by providers. As mentioned, it is unfortunate no information is available on the population which was seeking care/antibiotic use at the included venues, as this could be a key part in any stewardship interventions. Any intervention needs to include both the supply and demand challenges and drivers, which is also clear from several of the challenges mentioned which related to the knowledge, attitudes and practices of customers, without having insight in their perceptions. The need for a holistic approach could be reflected upon in the discussion and future directions as part of the related limitations already mentioned.

We agree that solely relying on our single study which focuses on the supply side is insufficient to arrive at a comprehensive set of recommendations. This is now clearly stated among the limitations, specifically:

> *"To verify the findings of this study, future work should elucidate both the supply- and demand-side drivers of antimicrobial use."*

Finally, even though the authors explained that the NYU Accra campus affirmed that IRB approval from NYU Abu Dhabi would be sufficient, it remains a serious concern that this potentially sensitive study did not request for IRB approval from Ghanian authorities. For most international research, IRB approval is sought in the country where data are collected, as well as in the country of origin of the main researchers. Are the Ghanian authorities aware?

NYU Accra and NYU Abu Dhabi are part of the same institution, NYU Global Network of campuses and sites, and IRB has been overseen and granted by NYU Abu Dhabi (given the affiliation of the researchers). The researchers have acted in good faith, and sought guidance on how to proceed, and followed the recommendations of the IRB Office. While seeking approval in

retrospect for a study is not possible, we agree with the Reviewer that a different consideration should be given in future studies. Our university's IRB Office determined the risk-level of the study and approved the study, with knowledge of the context within which the study would be undertaken. We however agree with the Reviewer's position, and have as such contacted our IRB Office and made them aware of the Reviewer's concern. We thank the Reviewer and hope that this communication will inform future practices.

Reviewer #4: • What are the main claims of the paper and how significant are they for the discipline?

The paper claims to investigate the challenges faced by medicine dispensers in Ghana from the perspective of the medicine vendors themselves. The topic was interesting as was the approach and the socio-ecological conceptual framework. The key challenges identified by the different types of medicine vendors included financial challenges faced by customers to pay the full cost of medicines, challenges in the treatment of illnesses like the vendors' knowledge and skills and lack of diagnostics, and broader health system challenges of a fragmented health system. These findings are all very well known in the wider literature from Africa and from other parts of the world including South and South East Asia. I felt that there were several gaps in the analysis due to which the authors have not been able to distinctly and persuasively present the novelty of their findings or highlight how their novel approach has revealed a fresh set of insights. So, it's difficult to justify that this paper makes a significant contribution to the existing literature on community based dispensing of medicines and healthcare.

We agree with the Reviewer that challenges faced by health systems in resource-limited settings, and with respect to dispensaries are well examined. We sought to expand the existing knowledge, and as the Reviewer rightly stated, by employing an often neglected approach, including our emphasis on the socio-ecological model of public health framework. We also note that through our review of the literature, we did not identify comparable studies that achieved the depth or breadth of our sample, and in particular, of medicine shops in Accra. In addition, prior studies often relied on anecdotal evidence with only a handful of shops involved, and were outdated. For instance, the most comparable work that we could identify had been published in the late 1990s. We believe that our context adds a different dimension to what is already known from the literature. We see our approach as novel by focusing on the perspectives of medicine vendors to examine their perspectives within a holistic framework. We believe that we document a shift in terms of AMR awareness among medicine sellers, how these shifts exist and operate at multiple levels, and the extent to which this multi-factored understanding should influence future interventions. We believe that our approach and findings have not been systematically discussed in the literature. Our study therefore contributes and advances our understanding of challenges encountered by medicine dispensaries in Accra and similar contexts, and could lead to improvements across multiple settings.

• Are the claims properly placed in the context of the previous literature? Have the authors treated the literature fairly?

There are ample references to previous literature, more than a hundred in fact, which seems rather excessive. Most are from Africa. I think the number of references could be reduced to about

60 and used more effectively and impactfully to make key points. It would also have been useful to bring in literature from a few countries like India and Bangladesh where there is a large community based informal sector that provides medicines including antibiotics at first contact. (See Gautham, M., et al., What are the challenges for antibiotic stewardship at the community level? An analysis of the drivers of antibiotic provision by informal healthcare providers in rural India. Social Science & Medicine, 2021. 275: p. 113813.)

We appreciate these points raised by the Reviewer. We have now carefully looked through the references to focus works cited, without overlooking key articles. We thank the Reviewer and have also included the suggested paper. Our strategy has been to stay geographically close when selecting the relevant literature, but indeed, the Reviewer raised a good point to expand this scope. Therefore, we have now included a number of papers in different contexts as well in addition to the specific suggestion of the Reviewer, for example, Do et al., 2021; Lam et al., 2021; Gyssens and Wertheim, 2020.

Many of the results sections begin with references to key studies that have shown similar findings. This use of literature in the results section was unnecessary as the results section should focus only on presenting the key findings, not on introducing them or discussing them vis a vis other studies. And I feel some key aspects in the literature have been missed out while making recommendations – for example, rapid diagnostics for antimalarials has been found to lead to unnecessary use of antibiotics in Africa, implying that there could be unintended consequences of diagnostics.

We appreciate the Reviewer's perspective, and believe that some of the literature cited in the Results section is well-placed. We were intentional in areas where we cite literature, and do so to remain grounded in contextual awareness. We have also looked into unintended consequences that the Reviewer suggests, and we have now added insight from Msellem and colleagues (2009) where we write: "*Note, however, that a replacement effect may occur, in which distributors default to antibiotics for cases with negative malaria test results as in [62].*"

Do the data and analyses fully support the claims? If not, what other evidence is required? I think the data needs to be analysed in greater depth and more critically in order to show the novelty of the findings and how these build on the existing literature. The key limitations are: -The differences between the trained pharmacists and the medicine vendors need to be brought out more clearly with respect to their profiles as well as their responses. Did they face similar challenges or were there major differences?

We appreciate this point, and we revisited our analysis. Returning to the literature we adjusted our categorization, which we believe already lends itself for a better analytical approach. In addition, we have now included a direct comparison of pharmacists and medicine vendors in chemical and herbal shops in Table 2, so that these two groups are comparable in terms of the challenges they mention. We have followed a similar approach when tabulating out demographic data (Table 1) about the sample to bring out what contrasts the two types of medicine vendors in terms of their background and working conditions as well.

-There are quite a few overlaps in the way the results are organized. For example, lack of diagnostics appears in organizational level, community level and public policy level, (but is missing in Fig 1). One way of presenting the sub themes under each level more clearly might be to provide clear sub-headings under each level, and make sure you only include the points relevant to that sub-theme under that sub-heading. The results are rather rambling at present and it is very difficult to understand what point you are trying to make. Before you submit a revised version, please do ask a couple of colleagues/friends who have not been involved in this paper to do an independent review and get their feedback.

We have significantly restructured the way in which our results are presented. We revisited our conceptual framework and modified the categories to be mutually exclusive. We then re-analyzed our data, using these more streamlined subcategories. With this in mind, we completely rewrote the Results section to be more clear. We hope that the Reviewer will agree with our assessment that the revised manuscript is much improved, and that the comprehension of the Results section has been significantly enhanced.

-You need to present the quotes in the appropriate style of a qualitative manuscript which is not the case at present. Only small quotes can be integrated into the text but the larger ones need to be presented separately, below the text, and the source needs to be added (anonymised but with an ID number and role). Please see the sample paper I have referenced earlier.

We have made these stylistic changes by adding respondent characteristics systematically with the quotes, such as age, gender, shop type and neighborhood. We refrained from adding IDs as they appeared idiosyncratic, and since this was not an interview study, we did not assign pseudonyms to respondents. To keep consistency, quotes are presented as part of the text as the overwhelming majority of them are short, one-sentence answers.

- You have skipped questions related to antibiotics for the non -pharmacists as they are not legally allowed to sell antibiotics. This is a HUGE limitation and must be acknowledged upfront because it severely limits what inferences you can draw about their antibiotic use. Perhaps you should limit this paper only to the qualified pharmacists.

We have now included information about this upfront, and repeat it in key places. However, we do not believe that the paper should be limited to pharmacists. We were able to discuss antimalarials with both pharmacists and medicine sellers, and our results affirm that these two sales follow similar patterns. We write:

> "*The Qualtrics questionnaire automatically skipped questions about antibiotics for chemical shops, to investigate only the over-the-counter medicines they were legally licensed to sell. While this presents a limitation, it was essential to minimize participation risks for the respondents.*"

We also write:

> *"To minimize the risk to subjects, we did not explicitly ask about illegal antibiotic distribution. Thus, inferences were made and extrapolated from available responses, such as questions focused on over-the-counter antimalarials. Although this is a clear limitation,*

it is crucial to note that respondents in herbal shops or chemical shops did not bring up self-medication or self-diagnosis as a major challenge that they navigated. This gives us confidence that this limitation is unlikely to mask important issues that we were unable to uncover.”

-The discussion should not repeat the findings but explain and discuss their implications. The last three recommendations are disconnected with the key findings. Another concern I have is that you have no policy recommendations for such a fragmented healthcare scenario.

We believe that the Discussion section should start with a summary of the key findings. To the Reviewer’s remark, however, we reviewed our summary of the overall findings to ensure that it is in a much streamlined fashion, and not simply a repetition.

Additionally, we have carefully considered the link between findings and recommendations. As pointed out by the other Reviewer we acknowledge that on the basis of this single study no comprehensive policy recommendations could be developed, however, suggestions need to be considered together with other complementary findings, e.g., the patient or demand side should be considered. We hope that the tentative suggestions, both policy and practice-related (stated in the Recommendations subsection) and the discussion of limitations together make for a more compelling rounding off of our paper, even if comprehensive policy recommendations are not put forward.

• If the paper is considered unsuitable for publication in its present form, does the study itself show sufficient potential that the authors should be encouraged to resubmit a revised version? Yes, I think that with an improved analysis and significantly improved presentation of the findings, the authors should be encouraged to resubmit a revised version.

We appreciate the confidence placed in our study, and hope that the revised manuscript lives up to these expectations.

• Are details of the methodology sufficient to allow the experiments to be reproduced?

No. It is not clear who administered the survey, and what probing was done to elicit responses to the question on challenges. Was the conceptual framework used to shape your questions, or was it used only later to guide the analysis? What was the total number of shops and pharmacies that you mapped? Was it 89? Can you provide a distribution of these by type of shop.

We have more clearly explained the methodology, which consisted of a 72-question survey deeply examining the practices of the given shop. The first 71 questions served as probes to investigate the context of practices and verify findings of the final question, where challenges were explicitly investigated. The survey itself is available as Supplement 1, which we hope can clarify some of the questions on the depth of the survey.

89 shops were visited, and 82 agreed to take part. Ultimately, full surveys were completed with 79 shops (35 herbal/chemical shops, and 44 pharmacies, as listed in Table 1). The ones that were incomplete were not included in the analysis.

We additionally reflected on the interviewers, and determined that there were no differences in response rate, and we believe the content of responses, between the two fieldworkers. One of them (HG) conducted 43 surveys while the other (RA) conducted 39 surveys.

• Is the manuscript well organized and written clearly enough to be accessible to non-specialists?

In general I found the introduction, methods and conceptual framework well organised and well written but the results and discussion need a lot more work to be clear and articulate for both specialist and non-specialist readers. Please see my specific comments in the attached word doc.

We appreciate the specific suggestions outlined by the Reviewer. We responded to these point-by-point and made changes accordingly. We also agree with the broader point, and our revision concentrated on streamlining the Results and Discussion sections the most, but we also made changes to the Introduction to make it tighter and more linearly structured.

Additional comments from Reviewer 4 (supplied in an attachment)

This paper is based on a useful and interesting study of community pharmacies and medicine shops in Ghana and follows a novel approach and conceptual framework to understand the challenges as articulated by the respondents themselves. In general, I found the introduction and methods clear and well written, but there are several gaps and weaknesses in the data analysis and presentation of the results, and in the discussion of the key findings. There is a lot of repetition and overlap in these sections and unnecessary use of references in the results section. The writing is somewhat rambling and jumpy and in some parts it is difficult to understand what point the authors are trying to make. Quotes need to be presented in the correct style of a qualitative report and with their source added (anonymised). Please see my specific comments below and I hope they will help you present your findings and your arguments more articulately:

Methods

Page 4, line 88: Did you use any further probes for this question, based on the socio-ecological conceptual framework?

This was the final question of the survey, and thus built upon the first 71 questions of the survey which were more nuanced and detail-oriented. The open-ended nature of this question sought to make space for participants to synthesize their prior responses and express any concerns that they wished to convey to researchers. The bulk of the survey delved specifically into patient demographics, sales, details of antimicrobials sold, and other related questions. This probing question sought to capture the breadth of shopkeeper experiences and thus prime the discussion of challenges.

Page 5, line 109: Was this an exhaustive mapping, covering every single shops in the city? Would be interesting to know how many shops were mapped in total and their breakdown by pharmacies

and chemical shops and herbal shops, just to understand the universe from which your sample was drawn. Are there more chemical shops than pharmacies in the universe?

We did not undertake a census of dispensaries in Accra, and cannot report on this based on our data collection effort. We also determined that very few have web or map presences, which made it challenging to confidently ascertain accurate numbers or even projections. It is important to note that in the months prior to survey administration, every single shop that was seen was saved as a pinned location, and all of these 89 saved locations were then visited for the purposes of collecting data. As noted, not all neighborhoods were completely "walked," and some neighborhoods fell outside of the scope of data collection. Therefore, beyond the 89 shops whose details were mapped on ethnographic visits, the study misses many others. Given the lack of census, it is hard to tell if pharmacies or chemical/herbal shops are more numerous.

Page 5, line 118-119: Why did you decide to leave out questions on antibiotic use for the chemical shops?? Since it is well known that these shops stock and sell antibiotics and many other non OTC drugs. This is a major limitation.

It is illegal for chemical shops to sell antibiotics, notwithstanding that these shops stock and sell antibiotics. We therefore decided to not ask herbal and chemical shops about illegal sales of medicines, and asked related questions to pharmacies. Importantly, ethical approval was granted by assessing the risks to participants, and the IRB Office determined that asking about illegal sales would pose shop owners/medical counter assistants at more than minimal risk encountered in everyday life. A way of protecting both the respondents and the data collection from legal challenges, these questions could not be asked outside of pharmacies. Herbal and chemical shops would be unlikely to take part in a survey if we asked about illegal drug distribution practices (which they would have needed to be aware of upfront in the beginning of the consent process), and we did not wish to risk bringing them any harm. In order to achieve these goals we did not ask about antibiotics if there was a chance that we elicited information of illegal activity.

Page 6, line 156: which version of Stata?

We previously used Stata version 18. The data analyses have been now carried out in R, and the manuscript reflects this change.

Results:

Page 6, lines 159-194: this is all utterly confusing and difficult to comprehend, especially as all through the intro you have talked about sites – pharmacies and shops - and here you start presenting the data by individual respondents, and lump them all together. I think if you present this data by the types of facilities it would be clearer.

We thank the Reviewer for this comment, and have added a table of Results broken down by shop type for clarity.

Show us in the tables who were the respondents (by their qualifications) in the pharmacies, chemical shops and herbal shops, and present their details clearly in the text.

We appreciate the suggestion and this has now been clarified, see Table 1.

Page 6, lines 185 – 189: please move all background information about qualifications and training and current regulations in the methods section or the intro.

For enhanced clarity, we have removed the parts that are not directly relevant to the challenges reported by participants. Specifically, we discuss qualifications in the “Study setting and context” subsection.

Page 7, Table 1: Can you provide these characteristics by different provider types. You could combine some of the provider categories and place them in a horizontal row.

We have changed the table to be broken down by shop types as requested.

Page 8, Table 2: This is again very confusing as you are now presenting results by the type of shops. What is the point of having this table? I assume in pharmacies you would have interviewed both pharmacists and lesser qualified attendants, and it would be interesting to know how these might differ. It’s important to be consistent in how you present the data.

We have removed and replaced the table with one broken down by shop types, as requested. However, we conducted one interview in each shop, so we do not have an opportunity to compare different respondents within the same shop.

Page 8, lines 226 – 232: Please move all this to the discussion.

We have moved it to the Introduction where the subthemes are explained.

Page 8, lines 232-234: These are important sub-themes. Please present them clearly and elaborate on each.

We hope that the updated model and streamlined writing addresses this comment.

Page 9, 238-239: this is not the right style of presenting quotes. Please read some qualitative papers and see how quotes are presented below the text and anonymised sources are provided.

We have changed the style of quotes to credit respondents by describing them demographically and place them in neighborhoods by neighborhood income.

Page 9, 243 – 244: again mixing up results and discussion. Results section should only include the findings.

We hope that the updated model and streamlined writing addresses this comment.

Page 9, 255-262: Very confusing. What is the point you are trying to make? Can you rewrite this in 2 lines please.

This has been revised as requested, and we hope that it properly addresses the Reviewer’s concern.

Page 10, 272-284: again mixing up background literature and discussion points with your findings. Instead of doing this, you can better utilise this space by elaborating on the findings in greater depth.

We have ensured that each section of the manuscript is concise and focused on the section of interest. We also ensure that the existing literature, when referenced in the discussion section, is directly in support of a point that we sought to make.

Please use sub-headings in the results sections so that you can clearly and distinctly describe the emerging sub-themes without repetitions.

We have added subheadings to improve clarity.

Page 12, 405-413: Delete this background info and use it in the discussion.

We have addressed this comment by moving the background information to the relevant sections of the manuscript, including the Discussion section.

Page 13, 331: This was a really interested point and a novel one which could have been elaborated much more – that people see them as doctors but they are not and therefore its risky to make a mistake. Unfortunately, you have turned this abruptly (and in a disconnected way) into a comment about their knowledge of brands of medicines. Please try to develop the previous point in a coherent way. And perhaps this fits better under the individual level.

We agree with the Reviewer that this finding is interesting and adds a dimension that has not been identified or explored in existing studies. This important finding, and broader theme has now been further elaborated in the manuscript. We caution that without further systematic probing on how respondents felt they were viewed, we are reluctant to elevate this point further than what we have included in the revised manuscript.

Page 13, 461- 464: Without their sources, the quotes are meaningless. Add the source as for example, pharmacist A1 in a pharmacy or pharmacy attendant B1 in a pharmacy, or medicine vendor C1 in a chemical shop

We have now added descriptions of respondents for each quote.

Page 14, 493-499: Please delete all background references.

We have moved this to the introduction section to explain each category.

Page 15, 533-536: shouldn't diagnostics be covered under organization?

We have now revisited our analysis and proposed four categories instead of five. This change has allowed us to more clearly delineate the various challenges, including those that are mutually exclusive.

Page 15, 561 – 564: So what is the challenge here?

The sentence has been removed.

Page 15, 569-571: How are these public policy challenges? This section is really weak but is a hugely important one, so please go back to your data and look for any other findings that can be included here. Did they know anything about the legal regulations? Were there any instances of bribery and corruption by the regulators?

Respondents did not identify policy as an avenue that should be pursued, as a way to improve their practices. We have now adjusted our categories for analysis to take this into account. The highest-level category is now Global, which does not specifically denote public policy. But, it does allow us to make some statements about policy, and especially in those categories where policy challenges are referenced. We add that respondents generally reported adhering to legal regulations on the tiered prescription requirements, and there was no mention of bribery and corruption.

Discussion:

Page 16, 587-588: but you haven’t even asked directly about antibiotics. and you certainly don’t get this impression from their answers - they are sharing challenges like patient demand and payment capacity that influence their inappropriate antibiotic use. So how can you conclude that there is improved desire to safeguard medications than what’s been reported in previous literature?

We now add more specific examples of how respondents seek to practice stewardship, which we hope addresses this concern.

Page 16, 593-595: look at the lit from other countries that also points to the broader social, economic and commercial drivers and not just individual ones.

These drivers have been included and are discussed in the introduction.

Page 17, lines 666-668: Not clear what you are to communicate. Aren't these contradictory statements?

We have rewritten this section for clarity.

Page 18, 674-675: This is not coming out through your findings at all. Strict adherence????

We now seek to more explicitly clarify the gap between intent and action. We more clearly highlight examples of respondents explaining how they *wish* to practice strict adherence, but are limited by various factors. We have edited this passage to no longer claim strict adherence but rather suggest that shopkeepers expressed an intent of practicing stewardship.

Page 20: In the limitations section, please add the fact that you skipped questions on antibiotics with the chemical sellers.

This has now been mentioned where relevant, including the Limitation subsection.
