# Supplementary material for: Challenges in the distribution of antimicrobial medications in community dispensaries in Accra, Ghana
Source: PLoS One. 2024 May 29;19(5):e0281699. doi: 10.1371/journal.pone.0281699 (PMC11135707; doi:10.1371/journal.pone.0281699)
Supplement: Attachment — Submitted filename: Response to Reviewers _ PLOS ONE AMR.docx [file pone.0281699.s002.docx]

**Challenges in the distribution of antimicrobial medications in community dispensaries in Accra, Ghana -** PONE-D-23-02556 - PLOS ONE

**Response to requested edits:**

**Journal requirements**

- *Comment 1: Please ensure that your manuscript meets PLOS ONE's style requirements, including those for file naming. The PLOS ONE style templates can be found at*

[*https://journals.plos.org/plosone/s/file?id=wjVg/PLOSOne_formatting_sample_main_body.pdf*](https://urldefense.proofpoint.com/v2/url?u=https-3A__journals.plos.org_plosone_s_file-3Fid-3DwjVg_PLOSOne-5Fformatting-5Fsample-5Fmain-5Fbody.pdf&d=DwQGaQ&c=slrrB7dE8n7gBJbeO0g-IQ&r=Jvu75lNPPPKjsHfNBE9XyeLTmHrrLiHvR0NaBuqZhWE&m=mVVY_hORks24bLIpHpf-6oKAlhfXGJmAMdwl-kaqoL8AL9YhITSLTFI4RiJBi774&s=HdZqmplGJKVkT5e1RgCSn5kN5mCRjqOg--GePOSq8fA&e=) *and* [*https://journals.plos.org/plosone/s/file?id=ba62/PLOSOne_formatting_sample_title_authors_affiliations.pdf*](https://urldefense.proofpoint.com/v2/url?u=https-3A__journals.plos.org_plosone_s_file-3Fid-3Dba62_PLOSOne-5Fformatting-5Fsample-5Ftitle-5Fauthors-5Faffiliations.pdf&d=DwQGaQ&c=slrrB7dE8n7gBJbeO0g-IQ&r=Jvu75lNPPPKjsHfNBE9XyeLTmHrrLiHvR0NaBuqZhWE&m=mVVY_hORks24bLIpHpf-6oKAlhfXGJmAMdwl-kaqoL8AL9YhITSLTFI4RiJBi774&s=9jjfiMRpq5K6aKVNm0tSIZcENMs5SNrzjCJb9f9CqPo&e=)

**Response:** The file name for the figure and supplemental information were changed to be Fig1 and S1_File, as required. The file type of the figure was changed from .png to .tif, and was checked with the PACE system. In the author list, initials were added for each of the corresponding authors’ email addresses to indicate each author.

- *Comment 2: We note that the grant information you provided in the ‘Funding Information’ and ‘Financial Disclosure’ sections do not match. When you resubmit, please ensure that you provide the correct grant numbers for the awards you received for your study in the ‘Funding Information’ section.*

**Response:** Our funding information has been updated, and is quoted below.

“The research fieldwork was funded by the New York University Abu Dhabi Division of Social Science as an undergraduate thesis project. Subsequent data analysis and writing were funded by a grant from the Effective Altruism Infrastructure Fund (a3r6g000001Tac2AAC). The funders had no role in study design, data collection and analysis, decision to publish, or preparation of the manuscript.”

- ***Comment 3:*** *Thank you for stating the following in the Acknowledgments Section of your manuscript: “Above all, we thank the participants of this research who shared their experiences and generously shared time out of their workdays, going above and beyond to explain the landscape of public health through their eyes. This fieldwork would not have been possible without Rafia Abdul-Mumin, who conducted 40 fieldsite visits. Her insight and hard work were invaluable, and the success of the study is truly indebted to her. The fieldwork for this project was funded by the Social Sciences Division at NYU Abu Dhabi, and the publication was funded by a grant from the Effective Altruism Infrastructure Fund.” We note that you have provided funding information that is currently declared in your Funding Statement. However, funding information should not appear in the Acknowledgments section or other areas of your manuscript. We will only publish funding information present in the Funding Statement section of the online submission form. Please remove any funding-related text from the manuscript and let us know how you would like to update your Funding Statement. Currently, your Funding Statement reads as follows: “The research fieldwork was funded by the New York University Abu Dhabi Division of Social Science as an undergraduate thesis project (of HG)*

*(*[*https://nyuad.nyu.edu/en/academics/undergraduate/undergraduate-research/capstone.html*](https://nyuad.nyu.edu/en/academics/undergraduate/undergraduate-research/capstone.html)*). Subsequent data analysis and writing were funded by a grant received by HG (*[*https://av20jp3z.paperform.co/?fund=EA*](https://urldefense.proofpoint.com/v2/url?u=https-3A__av20jp3z.paperform.co_-3Ffund-3DEA&d=DwQGaQ&c=slrrB7dE8n7gBJbeO0g-IQ&r=Jvu75lNPPPKjsHfNBE9XyeLTmHrrLiHvR0NaBuqZhWE&m=mVVY_hORks24bLIpHpf-6oKAlhfXGJmAMdwl-kaqoL8AL9YhITSLTFI4RiJBi774&s=uSXVnjdu080rm5RFm6pb3XE7qgE6XH5Xyb1AuLwVbI4&e=) *Infrastructure Fund) from the Effective Altruism Infrastructure Fund (*[*https://funds.effectivealtruism.org/funds/ea-community).*](https://urldefense.proofpoint.com/v2/url?u=https-3A__funds.effectivealtruism.org_funds_ea-2Dcommunity-29.&d=DwQGaQ&c=slrrB7dE8n7gBJbeO0g-IQ&r=Jvu75lNPPPKjsHfNBE9XyeLTmHrrLiHvR0NaBuqZhWE&m=mVVY_hORks24bLIpHpf-6oKAlhfXGJmAMdwl-kaqoL8AL9YhITSLTFI4RiJBi774&s=f59jaSY8arqm6yRpsW03nwWrmNFnH_3o6pUtGvOe7hQ&e=)  *The funders had no role in study design, data collection and analysis, decision to publish, or preparation of the manuscript.” Please include your amended statements within your cover letter; we will change the online submission form on your behalf.*

**Response:** We have removed all funding-related information from the manuscript, and now all this information is reflected in the Funding Statement. The updated funding statement reads as follows: “The research fieldwork was funded by the New York University Abu Dhabi Division of Social Science as an undergraduate thesis project. Subsequent data analysis and writing were funded by a grant from the Effective Altruism Infrastructure Fund (a3r6g000001Tac2AAC). The funders had no role in study design, data collection and analysis, decision to publish, or preparation of the manuscript.”

Because the mentioned RA is listed as an author, we changed the acknowledgments to read as follows: “Above all, we thank the participants of this research who shared their experiences and generously shared time out of their workdays, going above and beyond to explain the landscape of public health through their eyes.”

- *Comment 4. “We note that you have indicated that data from this study are available upon request. PLOS only allows data to be available upon request if there are legal or ethical restrictions on sharing data publicly. For more information on unacceptable data access restrictions, please see* [*http://journals.plos.org/plosone/s/data-availability#loc-unacceptable-data-access-restrictions*](https://urldefense.proofpoint.com/v2/url?u=http-3A__journals.plos.org_plosone_s_data-2Davailability-23loc-2Dunacceptable-2Ddata-2Daccess-2Drestrictions&d=DwQGaQ&c=slrrB7dE8n7gBJbeO0g-IQ&r=Jvu75lNPPPKjsHfNBE9XyeLTmHrrLiHvR0NaBuqZhWE&m=mVVY_hORks24bLIpHpf-6oKAlhfXGJmAMdwl-kaqoL8AL9YhITSLTFI4RiJBi774&s=B5Wz7tiNjvFQ-jUDrWDUkydKCK73ai-403f8iwa4fbE&e=)*. In your revised cover letter, please address the following prompts: a) If there are ethical or legal restrictions on sharing a de-identified data set, please explain them in detail (e.g., data contain potentially sensitive information, data are owned by a third-party organization, etc.) and who has imposed them (e.g., an ethics committee). Please also provide contact information for a data access committee, ethics committee, or other institutional body to which data requests may be sent. b) If there are no restrictions, please upload the minimal anonymized data set necessary to replicate your study findings as either Supporting Information files or to a stable, public repository and provide us with the relevant URLs, DOIs, or accession numbers. For a list of acceptable repositories, please see* [*http://journals.plos.org/plosone/s/data-availability#loc-recommended-repositories*](https://urldefense.proofpoint.com/v2/url?u=http-3A__journals.plos.org_plosone_s_data-2Davailability-23loc-2Drecommended-2Drepositories&d=DwQGaQ&c=slrrB7dE8n7gBJbeO0g-IQ&r=Jvu75lNPPPKjsHfNBE9XyeLTmHrrLiHvR0NaBuqZhWE&m=mVVY_hORks24bLIpHpf-6oKAlhfXGJmAMdwl-kaqoL8AL9YhITSLTFI4RiJBi774&s=BqyDWi2iX8pS3A_-SGtGR-iBnoGMtgQBwuL-vUrjOIQ&e=)*. We will update your Data Availability statement on your behalf to reflect the information you provide.*

**Response:** The data are indeed available as required. As per our ethics approval by the Institutional Review Board that these data can be shared after the removal of the neighborhood information to ensure that participants may not be reidentified. It is now available via the ICPSR repository used as requested, and the URL at which the data is accessible is as follows: <https://doi.org/10.3886/E192062V1>

- *Comment 5. PLOS requires an ORCID iD for the corresponding author in Editorial Manager on papers submitted after December 6th, 2016. Please ensure that you have an ORCID iD and that it is validated in Editorial Manager. To do this, go to ‘Update my Information’ (in the upper left-hand corner of the main menu), and click on the Fetch/Validate link next to the ORCID field. This will take you to the ORCID site and allow you to create a new iD or authenticate a pre-existing iD in Editorial Manager. Please see the following video for instructions on linking an ORCID iD to your Editorial Manager account:* [*https://www.youtube.com/watch?v=_xcclfuvtxQ*](https://urldefense.proofpoint.com/v2/url?u=https-3A__www.youtube.com_watch-3Fv-3D-5FxcclfuvtxQ&d=DwQGaQ&c=slrrB7dE8n7gBJbeO0g-IQ&r=Jvu75lNPPPKjsHfNBE9XyeLTmHrrLiHvR0NaBuqZhWE&m=mVVY_hORks24bLIpHpf-6oKAlhfXGJmAMdwl-kaqoL8AL9YhITSLTFI4RiJBi774&s=gfUAsVUKJ48usDO3iKDRsjOOuGACYVA7VTCnVrJjLgg&e=)

**Response:** The ORCID iD of Dr. Frimpong, Corresponding Author, has been validated (0000-0002-3930-7072). The ORCID of other researchers can be added as 0000-0002-4849-0606 for Kinga Makovi, 0000-0002-3865-4566 for Hannah Camille Greene.

- *Comment 6: Please remove your figures from within your manuscript file, leaving only the individual TIFF/EPS image files, uploaded separately. These will be automatically included in the reviewers’ PDF.*

**Response**: Figures have been removed from the manuscript text.

**Reviewer #1:**

***Introduction:***

- ***Comment 1.*** *Lines 25 - 26: Authors should consider providing current data on NTDs and malaria. The references are old and may not give a true picture of current trends. It is hard to believe that Accra, being the capital of Ghana, will have many NTDs. The paper referred to actually talks about non-communicable diseases in Accra and not NTDs.*

**Response:** We agree and changed the information in these sentences, updating the references to more recent sources from 2020 and 2021 (the World Malaria Report 2021 and Ghana’s 2020 Master Plan for NTDs Programme). The relevant part of the paper now reads: “Despite progress towards the elimination of some diseases, Ghana still faces endemic diseases requiring antibiotics for treatment \cite{ghana_health_services_master_2020}. Malaria persists as a perennial and endemic disease in Accra, and Ghana is one of the eleven countries with the highest burdens of malaria globally \cite{world_health_organization_world_2021}..”

**Methods:**

- ***Comment 1****. Lines 65 - 67: Consider revising to "Medicine vendors are reported to be the primary source of medicine dispensation in Accra, Ghana, and the whole of West Africa". The current sentence gives the impression of ranking with Accra leading*

**Response:** We agree, and therefore, we replaced the sentence as recommended. The section now reads: “As the capital of Ghana, the coastal city of Accra serves as a cosmopolitan node in the network of human and capital flows throughout West Africa. Medicine vendors are reported to be the primary source of medicine dispensation in Accra, and these medicines supply networks branch throughout West Africa \cite{baxerres_distribution_2021, baxerres_introduction_2021}.”

- ***Comment 2a. Sampling:*** *- Authors should provide details of how the sampling of shops was done (sampling frame etc)*
- ***Comment 2b-*** *What were the regions of Accra considered (line 91)?*

**Response:** We have added more detailed information. We first stratified neighborhoods known by local reputation for having residents of low/medium/high socioeconomic status, and then selected multiple neighborhoods within each tier. All regions of the city of Accra were considered eligible, but convenience sampling yielded neighborhoods centrally located around the university campus and within driving distance of each of the researchers. We sought to include a variety of neighborhoods spanning the economic diversity of the city, and ultimately 17 different neighborhoods within Accra were included.

- ***Comment 2c*** *- Any details on distance from hospitals considered?*

**Response**: We start to explore this in the Community-level section of results, but this is indeed a valuable aspect that should be explored further. We found anecdotally that shops clustered around hospitals (for example, five shops were directly across the street from a major public hospital), but proximity to a hospital was only considered when it was mentioned anecdotally in the open-ended responses by participants. Some responses indicated the proximity of a given shop relative to other healthcare institutions as challenges, but we did not quantify the exact distance from hospitals. This would indeed be a valuable avenue for further exploration.

- ***Comment 2d-*** *Most pharmacies will have multiple employees. How were the interviewees selected? Was one interviewee selected in each shop?*

**Response:** Many shops only had one employee working at the time of the visit. When multiple employees were present, they would collectively nominate one based on who wasn't busy at the moment, was in the highest-ranking role, had worked there longest and most likely had the capacity to answer questions about trends over time. This information is now included in the manuscript in the “Definition of study sample” section as “When multiple employees were present, they would collectively nominate one who was available at the time of the visit, or who had the most experience and most likely capacity to answer questions about trends over time

- ***Comment 3:***  *Provide detailed descriptions of the different types of staff in the shops.*

**Response:** We added detailed explanations of each role where their frequency is reported in the Results section. For convenience, this new text reads: “Roles were self-reported, but the titles of Pharmacist or MCA require standardized prerequisite courses by the Ghana Pharmacy Council. Pharmacists are required to complete at least four years of study prior to becoming a licensed Doctor of Pharmacy. They may begin work while still a pharmacy student (n=1). A registered Medicine Counter Assistant would complete a six-month course prior to taking the Ghana Medicine Counter Assistants Certificate Examination. Pharmacy Technicians (n=3) would require Ghana Pharmacy Technicians Qualifying Exam. The Healthcare Assistant (n=1) reported a two-year training course. Other employee roles, such as shop owner or herbal employee, were self-designated titles that did not suggest licensing. Respondents with multiple qualifications reported the highest-ranking title. Shop owners may also have been licensed MCAs, for instance, but reported "shop owner" as their most salient title. ”

**Results**:

- ***Comment 1.*** *Line 176: Provide references for the previous studies*

**Response:** References have been added.

- ***Comment 2****. Lines 179 - 182: This is not clear. Where are these under the individual circle in figure 1?*

**Response:** Thank you for pointing this out. Lines 179-182 were meant to fall in the category “occupational challenges” of the individual level challenges, but we see how this was not clear and agree with the need to clarify this. We considered these issues (insufficient income, language barrier, boredom, and not “having enough time to counsel each patient”) to fall under the category “occupational challenges,” (n=8). We have now changed the wording to reflect this, by modifying the sentence to “Among the occupational challenges cited by respondents were their insufficient income (specifically listed as the biggest challenge by one), a language barrier with customers (n=7), boredom (n=1), and not “having enough time to counsel each patient” (n=1).”

- ***Comment 3.*** *Lines 183 - : Quantify "most", "many" etc with proportions*

**Response:** Thank you for noting this; we revisited the entire text to look for ambiguous terms such as “most” and “many,” and added proportions or percentages where needed. At line 183 we have now reorganized to ensure necessary terms are quantified.

Table 1:

- ***Comment 1.*** *What is the professional background of the shop owners? If authors are interested in ownership status then that should be considered separately. Ownership cannot be put under the same variable as professionals.*

**Response:** Along with a more detailed explanation of the different employee titles, we added the line “Respondents with multiple qualifications reported their highest-ranking title. Shop owners may also have been licensed MCAs, for instance, but reported “shop owner” as their most salient title, therefore, we are unable to capture multiple titles, which is a limitation of how this detail was measured.”

- ***Comment 2.*** *What is the basis for the staff categorization? What is the difference between the health care assistant and the MCA? Who is a herbal employee?*

**Response:** We have added an explanation to clarify each categorization. At the beginning of Results, after “Respondents included 17 pharmacists and 42 Medicine Counter Assistants (MCAs)”, we added an explanation of each title: “Respondents included 17 pharmacists and 42 Medicine Counter Assistants (MCAs). Roles were self-reported, but the titles of Pharmacist or MCA require standardized prerequisite courses by the Ghana Pharmacy Council. Pharmacists are required to complete at least four years of study prior to becoming a licensed Doctor of Pharmacy. They may begin work while still a pharmacy student (n=1). A registered Medicine Counter Assistant would complete a six-month course prior to taking the Ghana Medicine Counter Assistants Certificate Examination. Pharmacy Technicians (n=3) would require Ghana Pharmacy Technicians Qualifying Exam. The Healthcare Assistant (n=1) reported a two-year training course. Other employee roles, such as shop owner (n=11), manager (n=1), or herbal employee (n=1), were self-designated titles that did not suggest licensing or other forms of credentials (n=13). All were recorded when multiple titles were given, but some respondents with multiple qualifications may have reported only their highest-ranking title. Shop owners may also have been licensed MCAs, for instance, but reported ``shop owner'' as their most salient title; therefore, we may not have captured all titles, which is a limitation of how this detail was measured.”

- ***Comment 3.*** *Authors should clarify what missing gender means. It's not clear how gender was* missed when participants were visited.

**Response:** For these 4 surveys, it seems that researchers accidentally skipped over this question. They are, therefore, missing as a result of human error. Since this information relies on self-identification, the researchers have not filled in this information based on their perceptions. We added text in the caption under Table 1 “Some responses may be missing due to researcher errors or other fieldwork circumstances.” The caption below the table now reads “Percentages were rounded up to the tenths place and may sum to more than 100%. The total number of responses may differ by question because some respondents declined to answer particular questions. Some responses may be missing due to researcher errors or fieldwork circumstances.”

- ***Comment 4.*** *Authors should define the different categories under source of employment. It's not clear how source of employment will be unknown when handling a sample of < 100.*

**Response:** We added the explanation: “Regarding the source of employment, self-founded enterprises (n=11) were those in which the participant was the original owner; family businesses (n=14) were when respondents were working in a shop owned by other family members or were those that had been inherited from other family members. Those who reported “Referral” as source of employment (n=10) were those who had been given the job via other mutual connections. 31 reported having submitted a formal application. One replied that they had seen an advertisement and two had seen online listings. Six had completed internships in their location, which subsequently conferred the opportunity for formal paid employment. Five respondents chose not to reveal their origin of employment.”

**Table 2:**

- ***Comment 1.*** *"Year of starting to work in this location" should be separated from the rest of variables in the table. Mean year with SD etc appears misplaced. Authors should consider proportions of shopkeepers starting work in each of the given years (2014 - 2021) per type of shop keeper.*

**Response:** We agree that this information was misplaced, and deleted “Year of starting to work in this location” from the Table, because the variable “number of years working in this location” can already capture this information.

- ***Comment 2.*** *Generally, can authors relate results to type of shopkeeper interviewed in terms of role, age-group, gender, years of work etc. Will be interesting to know what the different types of shopkeepers are saying. This will inform any planned interventions.*

**Response:** We appreciate this suggestion and agree that this can significantly strengthen the paper.

We have added Table 3 to further highlight what we agree to be important differences by type of shop. We created Table 3 to show how the number of challenges reported in each tier of the socioecological model may vary across different shopkeeper demographics. We believe Tables 1 and 2 begin to address the important point that was raised about highlighting differences by type of shop as they emphasize substantive differences such as months of training, hours that the shop is open each day, etc., categorized by type of shop, and we believe that the readership of the journal would find this information useful. In addition, we have added another table (Table 3), to further emphasize differences. In Table 3, we show challenges reported by respondents, by type of shop, role of respondent, gender, and age.

**Reviewer #2:**

**Abstract:**

- ***Comment 1:*** *I recommend the removal of both hyphens in the sentence in the abstract which says: ‘Yet patterns in medicine distribution—and their consequences—are not well understood’.*

**Response:** Thank you very much for this careful review. We agree, this would improve the sentence, and have removed the hyphens as requested.

- ***Comment 2:*** *Secondly, I recommend elaborating on the methodology in the abstract section. The current information is skeletal and does not to provide sufficient information on methods applied. As I understand you may be constrained by the word count, I recommend shortening the introduction to achieve this.*

**Response:** We agree and modified the methodology line to be as follows: “Fieldwork was conducted in 80 medicine shops throughout Accra, using an in-depth survey questionnaire administered verbally. To determine the challenges faced by shopkeepers, responses to open-ended questions were analyzed using the socioecological model of public health.”

- ***Comment 3:*** *Thirdly, the authors state a two-fold objective. However, the results in the abstract appear to focus on only the first objective (challenges). I am wondering if authors identified opportunities for improving community-level stewardship of antimicrobials from the survey and I recommend including these in the abstract.*

**Response:** We modified the results section of the abstract to be more clear, as suggested.

Main Article:

- ***Comment 1:*** *I recommend the removal of hyphens in lines 10 and 11, and between words in other parts of the manuscript.*

**Response:** We have removed hyphens as requested.

- ***Comment 2:*** *Most importantly, I recommend a thorough review of the introduction to ensure there is a flow of ideas culminating in a comprehensive study rationale. The current introduction appears to consist of distinct parts with two study rationales in lines 20-23 and 58-59 making the research ideas appear quite disjointed. The introduction should be more concise and integrate both ideas. This should be followed by a comprehensive research rationale at the end.*

**Response:** Thank you for highlighting this; we agree that the flow needed to be improved, so we revisited the introduction and reordered the text to improve the flow of ideas, as suggested. These changes also made it possible to ground the design of the study in the literature better.

- ***Comment 3:*** *Lastly, the study was conducted in Ghana but approved by the New York University, Abu Dhabi Institutional Review Board. There is no mention of ethical approval with any Ghanaian institution. I am wondering if you such approval was obtained.*

**Response:** In the process of preparing for IRB approval at NYU Abu Dhabi, we discussed with the administration of the NYU Accra campus, who affirmed that the NYU Abu Dhabi-based IRB approval would be sufficient in line with international conventions on research approvals. We also relied on the expertise of our local faculty member from the NYU Ghana campus who aided in guiding the development of the survey, which he deemed appropriate and acceptable.
