# Supplementary material for: Challenges in the distribution of antimicrobial medications in community dispensaries in Accra, Ghana
Source: PLoS One. 2024 May 29;19(5):e0281699. doi: 10.1371/journal.pone.0281699 (PMC11135707; doi:10.1371/journal.pone.0281699)
Supplement: Attachment — Submitted filename: Reviwers comments.docx [file pone.0281699.s003.docx]

**Title: Challenges in the distribution of antimicrobial medications in community dispensaries in**

**Accra, Ghana**

This paper is based on a useful and interesting study of community pharmacies and medicine shops in Ghana and follows a novel approach and conceptual framework to understand the challenges as articulated by the respondents themselves. In general, I found the introduction and methods clear and well written, but there are several gaps and weaknesses in the data analysis and presentation of the results, and in the discussion of the key findings. There is a lot of repetition and overlap in these sections and unnecessary use of references in the results section. The writing is somewhat rambling and jumpy and in some parts it is difficult to understand what point the authors are trying to make. Quotes need to be presented in the correct style of a qualitative report and with their source added (anonymised). Please see my specific comments below and I hope they will help you present your findings and your arguments more articulately:

**Methods**

Page 4, line 88: Did you use any further probes for this question, based on the socio-ecological conceptual framework?

Page 5, line 109: Was this an exhaustive mapping, covering every single shops in the city? Would be interesting to know how many shops were mapped in total and their breakdown by pharmacies and chemical shops and herbal shops, just to understand the universe from which your sample was drawn. Are there more chemical shops than pharmacies in the universe?

Page 5, line 118-119: Why did you decide to leave out questions on antibiotic use for the chemical shops?? Since it is well known that these shops stock and sell antibiotics and many other non OTC drugs. This is a major limitation.

Page 6, line 156: which version of Stata?

**Results:**

Page 6, lines 159-194: this is all utterly confusing and difficult to comprehend, especially as all through the intro you have talked about sites – pharmacies and shops - and here you start presenting the data by individual respondents, and lump them all together. I think if you present this data by the types of facilities it would be clearer. Show us in the tables who were the respondents (by their qualifications ) in the pharmacies, chemical shops and herbal shops, and present their details clearly in the text.

Page 6, lines 185 – 189: please move all background information about qualifications and training and current regulations in the methods section or the intro.

Page 7, Table 1: Can you provide these characteristics by different provider types. You could combine some of the provider categories and place them in a horizontal row.

Page 8, Table 2: This is again very confusing as you are now presenting results by the type of shops. What is the point of having this table? I assume in pharmacies you would have interviewed both pharmacists and lesser qualified attendants, and it would be interesting to know how these might differ. It’s important to be consistent in how you present the data.

Page 8, lines 226 – 232: Please move all this to the discussion.

Page 8, lines 232-234: These are important sub-themes. Please present them clearly and elaborate on each.

Page 9, 238-239: this is not the right style of presenting quotes. Please read some qualitative papers and see how quotes are presented below the text and anonymised sources are provided.

Page 9, 243 – 244: again mixing up results and discussion. Results section should only include the findings.

Page 9, 255-262: Very confusing. What is the point you are trying to make? Can you rewrite this in 2 lines please.

Page 10, 272-284: again mixing up background literature and discussion points with your findings. Instead of doing this, you can better utilise this space by elaborating on the findings in greater depth.

Please use sub-headings in the results sections so that you can clearly and distinctly describe the emerging sub-themes without repetitions.

Page 12, 405-413: Delete this background info and use it in the discussion.

Page 13, 331: This was a really interested point and a novel one which could have been elaborated much more – that people see them as doctors but they are not and therefore its risky to make a mistake. Unfortunately, you have turned this abruptly (and in a disconnected way) into a comment about their knowledge of brands of medicines. Please try to develop the previous point in a coherent way. And perhaps this fits better under the individual level.

Page 13, 461- 464: Without their sources, the quotes are meaningless. Add the source as for example, pharmacist A1 in a pharmacy or pharmacy attendant B1 in a pharmacy, or medicine vendor C1 in a chemical shop

Page 14, 493-499: Please delete all background references.

Page 15, 533-536: shouldn’t diagnostics be covered under organization?

Page 15, 561 – 564: So what is the challenge here?

Page 15, 569-571: How are these public policy challenges? This section is really weak but is a hugely important one, so please go back to your data and look for any other findings that can be included here. Did they know anything about the legal regulations? Were there any instances of bribery and corruption by the regulators?

**Discussion:**

Page 16, 587-588: but you haven’t even asked directly about antibiotics. and you certainly don’t get this impression from their answers - they are sharing challenges like patient demand and payment capacity that influence their inappropriate antibiotic use. So how can you conclude that there is improved desire to safeguard medications than what’s been reported in previous literature?

Page 16, 593-595: look at the lit from other countries that also points to the broader social, economic and commercial drivers and not just individual ones.

Page 17, lines 666-668: Not clear what you are to communicate. Aren't these contradictory statements?

Page 18, 674-675: This is not coming out through your findings at all. Strict adherence????

Page 20: In the limitations section, please add the fact that you skipped questions on antibiotics with the chemical sellers.
